# High‐efficiency prime editing enables new strategies for broad‐spectrum resistance to bacterial blight of rice

**DOI:** 10.1111/pbi.14049

**Published:** 2023-05-03

**Authors:** Ajay Gupta, Bo Liu, Qi‐Jun Chen, Bing Yang

**Affiliations:** ^1^ Division of Plant Science and Technology, Bond Life Sciences Center University of Missouri Columbia Missouri USA; ^2^ State Key Laboratory of Plant Physiology and Biochemistry, College of Biological Sciences China Agricultural University Beijing China; ^3^ Center for Crop Functional Genomics and Molecular Breeding China Agricultural University Beijing China; ^4^ Donald Danforth Plant Science Center St. Louis Missouri USA

**Keywords:** prime editing, CRISPR/Cas9, *Xanthomonas*, rice, *xa5*, *xa23*

## Abstract

Using genetic resistance against bacterial blight (BB) caused by *Xanthomonas oryzae* pathovar *oryzae* (*Xoo*) is a major objective in rice breeding programmes. Prime editing (PE) has the potential to create novel germplasm against *Xoo*. Here, we use an improved prime‐editing system to implement two new strategies for BB resistance. Knock‐in of TAL effector binding elements (EBE) derived from the BB susceptible gene *SWEET14* into the promoter of a dysfunctional executor *R* gene *xa23* reaches 47.2% with desired edits including biallelic editing at 18% in T_0_ generation that enables an inducible TALE‐dependent BB resistance. Editing the transcription factor TFIIA gene *TFIIAγ5* required for TAL effector‐dependent BB susceptibility recapitulates the resistance of *xa5* at an editing efficiency of 88.5% with biallelic editing rate of 30% in T_0_ generation. The engineered loci provided resistance against multiple *Xoo* strains in T_1_ generation. Whole‐genome sequencing detected no *OsMLH1dn*‐associated random mutations and no off‐target editing demonstrating high specificity of this PE system. This is the first‐ever report to use PE system to engineer resistance against biotic stress and to demonstrate knock‐in of 30‐nucleotides cis‐regulatory element at high efficiency. The new strategies hold promises to fend rice off the evolving *Xoo* strains and protect it from epidemics.

## Introduction

Crop diseases cause significant yield loss and threaten food security globally, which will be worsened by climate changes (Velasquez *et al*., 2018). One major example is bacterial blight of rice (BB) caused by *Xanthomonas oryzae* pathovar *oryzae* (*Xoo*). Rice is a staple crop that provides food to about half of the world's population; however, BB disease alone can cause up to 70% of yield damage in case of severe infection (Srinivasan and Gnanamanickam, 2005). This makes BB resistance the most important target for rice genetic improvement programmes. *Xoo* uses various virulence strategies to colonize host plants and eventually precipitate yield loss (Zhang and Wang, 2013). The extraordinary strategy is that *Xoo* weaponizes its transcription activator‐like effectors (TALEs) to condition a state of blight susceptibility, for example using AvrXa7 and PthXo3 to activate *SWEET14* (Antony *et al*., 2010), PthXo2 and its variants to target *SWEET13* (Zhou *et al*., 2015), and PthXo1 to commandeer *SWEET11a* (Yang *et al*., 2006), all transcriptionally. These TALEs of virulence bind to the promoter sequences, so‐called effector binding elements (EBEs), of the host susceptibility genes (Oliva *et al*., 2019). The EBEs represent the vulnerability spots for pathogen to exploit host. On the contrary, host rice has evolved mechanisms to counteract the pathogenicity strategies. It has been discovered that some rice populations alter the disease‐vulnerable EBEs in the *SWEET* genes to make them unresponsive to TALEs, resulting in the genetic recessive resistance genes (Eom *et al*., 2019). Rice also co‐opts EBEs in the promoters of defence genes, so‐called executor *R* genes, to trap some TALEs for triggering BB resistance, for example *Xa23* corresponding to AvrXa23 (Wang *et al*., 2015). *Xa23* allele differs from its dysfunctional allele *xa23* in their polymorphic EBEs within their promoter regions (Wang *et al*., 2015). Rice has also evolved a BB resistance mechanism by mutating the *TFIIAγ5* gene that encodes the general transcription factor TFIIA gamma subunit by a single amino acid change (V39E), resulting in the recessive resistance gene *xa5* (Huang *et al*., 2016; Iyer and McCouch, 2004). It was later found that *xa5* reduces the abilities of several major TALEs to activate host susceptibility genes, leading to BB resistance (Huang *et al*., 2016).

Genome editing particularly with the engineered CRISPR‐Cas systems has advanced our basic understanding of life sciences and applications in medical field and agriculture. The CRISPR‐based genome editing systems depend on the abilities of Cas proteins and variants to introduce genomic DNA double or single‐strand cut when directed to a preselected target site by the programmable guide RNA. The DNA break and the cellular DNA repair process are exploited to achieve genetic modifications in the genome of interest. The most popular system is the CRISPR‐Cas9 for targeted mutagenesis due to its simplicity and high efficiency, but it has limitations for some applications such as desired nucleotide changes (Pickar‐Oliver and Gersbach, 2019). Prime editing (PE), therefore, has been developed to overcome those constraints. PE depends on a nickase Cas9 fused to a reverse transcriptase domain (nCas9‐RT) and a prime‐editing guide RNA (pegRNA). pegRNA comprises a guide for nCas9 and a 3′ extended template for RT. The guide directs nCas9‐RT to bind and nick genomic DNA, resulting in a single‐stranded DNA break that primes reverse transcription of template sequence. The newly synthesized DNA strand results in an extended 3′ flap that contains the desired nucleotides, which are subsequently incorporated into the genome via 5′ flap excision, ligation and heteroduplex resolution (Anzalone *et al*., 2019). The initial PE was less efficient and produced undesired insertion/deletion by‐products, and editing efficiency varies widely across different species, cell types, loci and edit sequences (Lin *et al*., 2020). However, great efforts have been made to improve its efficiency and precise small insertions and deletions (indels) as well as all 12 base conversions and reducing the levels of indel by‐products, thus offering greater versatility than other CRISPR nucleases. The major improvements include approaches to increasing pegRNA expression (Jiang *et al*., 2020), enhancing pegRNA stability (Nelson *et al*., 2022), engineering more efficient nCas9 and reverse transcriptase (Zong *et al*., 2022), and using the host DNA mismatch repair inhibiting protein (Chen *et al*., 2021).

With the knowledge of virulence and the counteracting resistance mechanisms in the bacterial blight of rice, scientists have engineered BB resistance by exploiting two strategies using genome editing. The first one is to use CRISPR‐Cas9 to mutagenize the EBEs in *SWEET* genes to achieve BB resistance (Oliva *et al*., 2019). The second strategy is to use CRISPR‐Cas9 and homologous recombination approach to insert the functional AvrXa23 EBE of the executor *R* gene *Xa23* in the dysfunctional promoter of *xa23*, enabling AvrXa23‐dependent BB resistance in the otherwise susceptible rice variety (Wei *et al*., 2021). In the latter case, homologous recombination approach was implemented to insert the AvrXa23 EBE of *Xa23* into the promoter of *xa23*, which is low efficient and needs co‐delivering of Cas9/gRNA and EBE‐containing donor DNA by biolistic bombardment (Wei *et al*., 2021).

In this study, we used an improved PE system, PE5max (Jiang *et al*., 2022), to engineer BB resistance through two new strategies based on the host counteracting mechanism against TALEs of *Xoo*. We first sought to create a functional *xa23* by inserting the 30‐bp long AvrXa7 and PthXo3 overlapping EBEs from the BB susceptibility (S) gene *SWEET14* into the promoter of *xa23* in rice, resulting in dominant *Xa23* alleles named hereafter *Xa23* ^*SW14*^ and second to create the V39E allele (*xa5*) of *TFIIAγ5* (Figure S1a,b). Both editing strategies produced high‐efficiency editing and bestowed strong BB resistance upon the otherwise susceptible rice. In both editing cases, more than 18% of lines were biallelically edited among the total edited lines in T_0_ generation. Moreover, the whole‐genome resequencing of edited lines revealed no evidence of off‐target editing or increased rate of random mutations due to the negative dominant mismatch repair protein OsMLH1dn, a component of PE5max system. This is the first case to our knowledge to utilize PE in plants for providing resistance against pathogen and the first case to report the successful insertion of relatively large DNA elements (30‐bp) at a very high efficiency in plants.

## Results

### Strategy 1: Prime editing efficiently inserts effector binding element (EBE) to the promoter of dysfunctional executor gene *xa23* and generates dominant resistance gene *Xa23* ^*SW14*^

**Figure 1:**
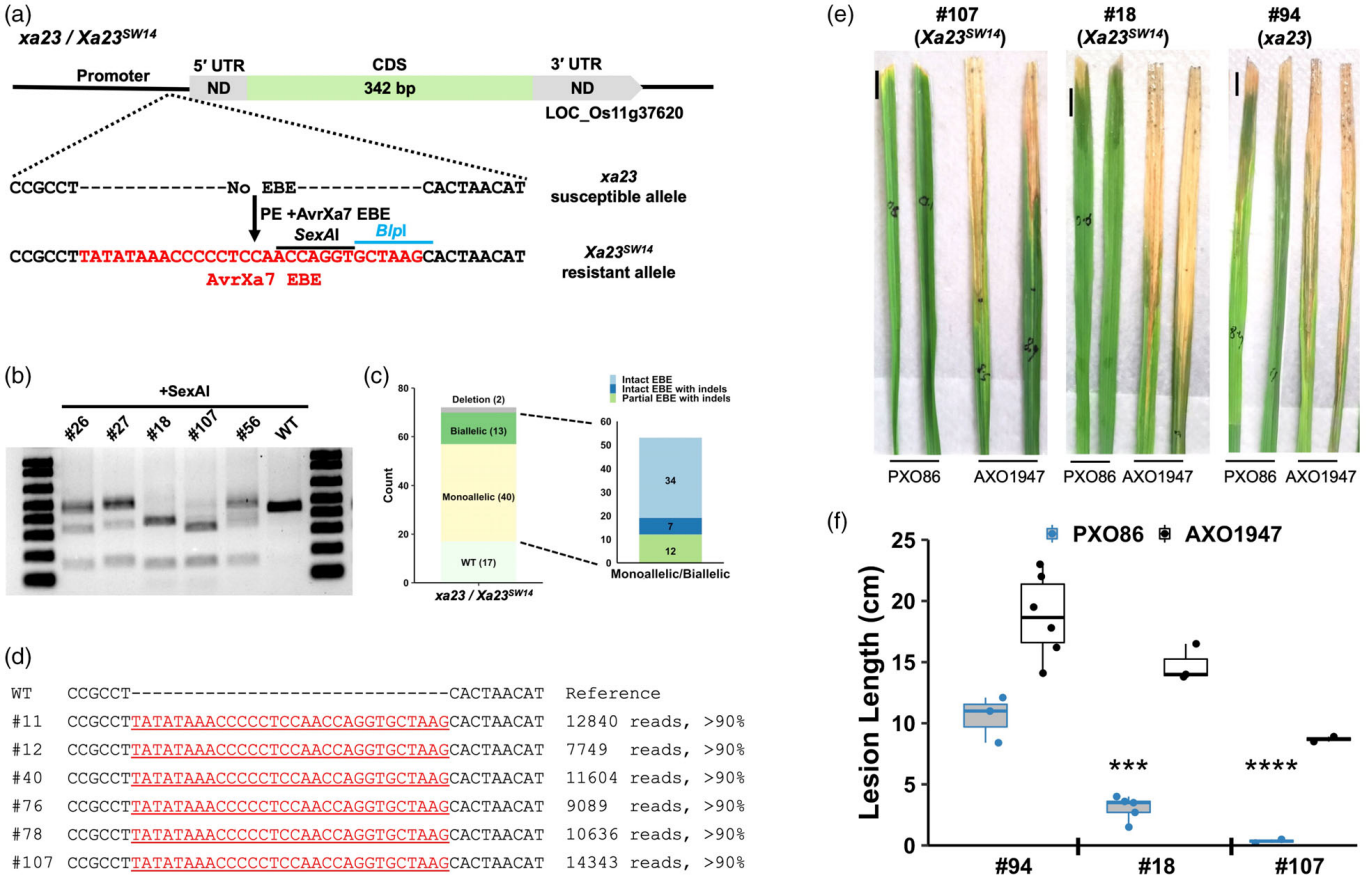
PE‐based knock‐in of EBE in *xa23*. (a) Gene structures of *xa23* and *Xa23* ^*SW14*^. Intronless CDS and untranslated sequences (ND, not determined) are shown. Intended knock‐in sequence of EBEs is in red, with restriction sites shown. (b) Gel image showing *SexA*I cleavage of relevant PCR products of some representative T_0_ and the wild‐type (WT) lines. (c) Summary of editing events based on gel and deep sequencing of PCR amplicons. Complete, partial digestions and undigested lines are diallelic, monoallelic edits and wild type, respectively. Number in parenthesis shows the count out of total T_0_ lines. The second bar illustrates the types of edits occurred in monoallelic and diallelic lines based on the deep amplicon sequencing of *xa23*. (d) Deep sequencing of *xa23/Xa23* ^*SW14*^ amplicons. Independent biallelic lines with intact EBE (in red) inserted without indels are shown. Total number and percentage of reads of individual edited lines are presented on the right. Dashes in reference separate the two bases where EBE is inserted. (e,f) Lesion lengths of two biallelic and a wild‐type line inoculated with PXO86 and AXO1947. Lesion lengths were measured 12 days postinoculation on three to five leaves of individual plants (*n* = 3–5). Scale bar, 1 cm. ***P‐value <0.001 and ****P‐value <0.0001 with *t*‐test adjusted using Bonferroni correction for multiple comparisons.

Most rice lines including Kitaake have a dysfunctional executor gene *xa23*, which differs from its functional counterpart, *Xa23*, in the promoter region. Kitaake was chosen for this study due to its few known BB resistance genes, allowing us to test the strategies for engineered resistance in edited rice lines. The *Xa23‐*harbouring rice line IRBB23 has an EBE trap in its promoter corresponding to *Xoo* TALE AvrXa23 (Figure S2). The *xa23* allele has been demonstrated to provide strain‐specific resistance when activated via designer TALE and HR‐based insertion of AvrXa23 EBE in its promoter (Wang *et al*., 2017; Wei *et al*., 2021). *SWEET14* is a sucrose transporter gene and the susceptibility (*S*) gene targeted by AvrXa7 and PthXo3, which are two TALEs present in the majority of Asian *Xoo* isolates (Oliva *et al*., 2019). Broad‐spectrum BB resistance might, therefore, be obtained by introducing the 30‐bp long overlapping EBE region from *SWEET14* into the promoter of *xa23* (Figure 1a and Figure S1b). The PE construct with the EBEs incorporated in the pegRNA was used for *Agrobacterium‐*mediated rice transformation. Seventy‐two transgenic events were obtained and genotyped with relevant PCR products against *SexA*I and *Blp*I, arisen after successful EBE knock‐in. Out of the 72 lines, 13 biallelic, 40 monoallelic, 17 wild‐type and two deletion lines were identified (Figure 1b,c). We then deep‐sequenced the PCR amplicons of all biallelic and monoallelic lines (*n* = 53) to capture the entire spectrum of edits. Out of the 53 sequenced lines, 34 had intact EBE insertions without any by‐products, seven had intact EBE insertion combined with indels at the pegRNA or the nicking gRNA (ngRNA) target site, and 12 lines had partial EBE sequences coupled with indels at the pegRNA or ngRNA target sites (Figure 1c,d and Table S3). Overall, a high percentage of edited lines (73.6%, *n* = 53) was recovered, including 47.2% (*n* = 34) lines carrying desired EBE insertions and 18% (*n* = 13) lines containing biallelic edits (Figure 1c,d and Table S3). We then determined the inducibility of edited *xa23* (referred to as *Xa23* ^*SW14*^) by syringe infiltrating leaves of two independent biallelic lines with *avrXa7*‐harbouring strain PXO86 and *avrXa7*‐lacking strain AXO1947 and performed RT‐PCR on the cDNA. As expected, both biallelic lines displayed a strong *Xa23* ^*SW14*^ induction by PXO86 but not by AXO1947 (Figure S3a,b). When assessed for disease response, both lines were highly resistant to PXO86 and susceptible to AXO1947 compared with wild‐type line (Figure 1e,f). The findings indicate that PE can be utilized to generate novel TALE‐dependently inducible *R* alleles by the promoter EBE trap strategy at high efficiency.

### Strategy 2: Prime editing efficiently generates recessive *xa5* resistant allele in T_0_ generation

**Figure 2:**
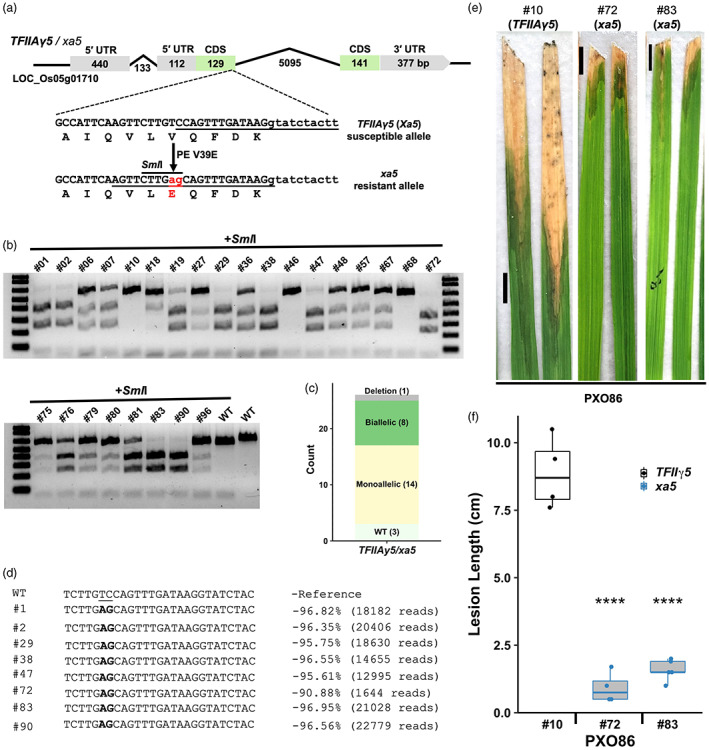
Prime editing of *TFIIAγ5* to generate *xa5* allele. (a) Gene structures of *TFIIAγ5* and *xa5*. PE target site (underlined) in *TFIIAγ5* allele and nick site (underlined) in the edited strand of *xa5* allele are shown. (b) Genotyping of edited lines as indicated above lanes with *Sml*I digestion of relevant PCR amplicons. (c) Bar plot summarizing the *Sml*I digestion. Complete, partial digestions and undigested lines are diallelic, monoallelic edits and wild type, respectively. (d) Deep sequencing of biallelic lines with desired edits. The wild‐type bases are underlined in the reference sequence and edited bases are bold highlighted in sequenced lines. The numbers on the left indicate the percentage and number of reads mapped to the *xa5* allele. (e,f) Disease phenotypes of edited biallelic lines. Lesion lengths were measured 12 days post inoculation with PXO86 on three to five leaves of individual plants (*n* = 3–5). Scale bar, 1 cm. **** represents *P*‐value <0.0001 with *t*‐test adjusted using Bonferroni correction for multiple comparisons.

The *xa5* gene is effective against *Xoo* strains that depend on the major TALE genes *avrXa7*, *pthXo3*, *pthXo2* variants, *TalC* and *TalF*. However, the *xa5* allele is rare in rice cultivars (at 3.14%), occurring most commonly in *aus* rice among the 3017 varieties in the 3 k rice database (Mansueto *et al*., 2017). Since the rice recessive resistant allele *xa5* differs only at one amino acid position (V39E) from its dominant susceptible counterpart *TFIIAγ5* to provide broad‐spectrum resistance against *Xoo*, we decided to edit Kitaake to generate this allele using the PE5max system (Figure 2a). The PE construct was used for *Agrobacterium*‐mediated rice transformation.

Twenty‐six independent transgenic events were recovered and genotyped for the V39E editing based on *Sml*I restriction sequence arisen from successful editing (Figure 2a). Out of the total 26 T_0_ lines, 23 showed cleavages with *Sml*I indicative of an editing efficiency of 88.5% (Figure 2b). Out of these 23 edited T_0_ lines, eight were biallelic (edits in two alleles) at 30.7% and 14 monoallelic (only one allele edited) at 57.8% as evidenced by complete and partial *Sml*I cleavage, respectively, while one had a large deletion (Figure 2b,c). To further determine occurrence of desired edits, unintended by‐products or guide RNA scaffold‐associated base changes, we deep‐sequenced the PCR amplicons of all biallelic lines using Illumina paired‐end reads. The results showed that all the T_0_ biallelic lines had desired V39E edits, and no undesired changes were detected including indel mutations and by‐products derived from the pegRNA scaffold (Figure 2d). Next, several of the T_0_ biallelic, wild‐type and a few monoallelic lines were challenged against an *avrXa7‐*containing *Xoo* strain PXO86. Indeed, the T_0_ *xa5*‐Kitaake plants were highly resistant to PXO86 in all biallelic lines tested compared with the wild‐type and monoallelic counterparts (Figure 2e, f and Figure S4a,b), while the same lines were susceptible to the *pthXo1* containing *Xoo* strain PXO99 (Figure S4a,b). The major TALE effector *pthXo1* remains virulent and can cause disease even in the presence of *xa5* (Huang *et al*., 2016). The results demonstrate that the engineered *xa5* allele is functional and recessive like the natural *xa5* allele and that this strategy and technology can be utilized to engineer *xa5* in elite rice cultivars without conventional breeding strategies.

### PE‐generated alleles *Xa23^SW14^* and *xa5* are heritable and provide broad‐spectrum resistance.

One major aspect of genome editing is the heritability of edits into subsequent generations. To test the heritability of newly generated *Xa23* ^*SW14*^ and *xa5* alleles, we selected four biallelic edited lines and one WT control line each from the two T_0_ populations to grow the T_1_ generation. As expected, all the tested biallelic lines were homozygous for *Xa23* ^*SW14*^ and *xa5* while the WT control lines were homozygous for *xa23* and *TFIIAγ5* alleles as genotyped using PCR‐RE with *Blp*I for *Xa23* ^*SW14*^ and *Sml*I for *xa5* in T_1_ generation (Table 1).

**Table 1:** Heritability of T_0_ edits to T_1_ generation

Gene	T_0_ line	T_0_ genotype	# Of plants	Genotype (# of plants)		
				*xa23/xa23*	*xa23*/*Xa23* ^*SW14*^	*Xa23* ^*SW14*^/*Xa23* ^*SW14*^
*xa23/Xa23* ^*SW14*^	#11	*Xa23* ^*SW14*^ */Xa23* ^*SW14*^	24	0	0	24
	#18	*Xa23* ^*SW14*^ */Xa23* ^*SW14*^	22	0	0	22
	#40	*Xa23* ^*SW14*^ */Xa23* ^*SW14*^	22	0	0	22
	#94	*xa23/xa23*	24	24	0	0
	#107	*Xa23* ^*SW14*^ */Xa23* ^*SW14*^	22	0	0	22

Gene	T_0_ line	T_0_ genotype	# of plants	Genotype (# of plants)		
				*TFIIγ5/TFIIγ5*	*TFIIγ5/xa5*	*xa5/xa5*
*TFIIγ5/xa5*	#1	*xa5/xa5*	24	0	0	24
	#2	*xa5/xa5*	24	0	0	24
	#38	*xa5/xa5*	19	0	0	19
	#47	*xa5/xa5*	24	0	0	24
	#68	*TFIIγ5/ TFIIγ5*	15	15	0	0
	#83	*xa5/xa5*	17	0	0	17

**Figure 3:**
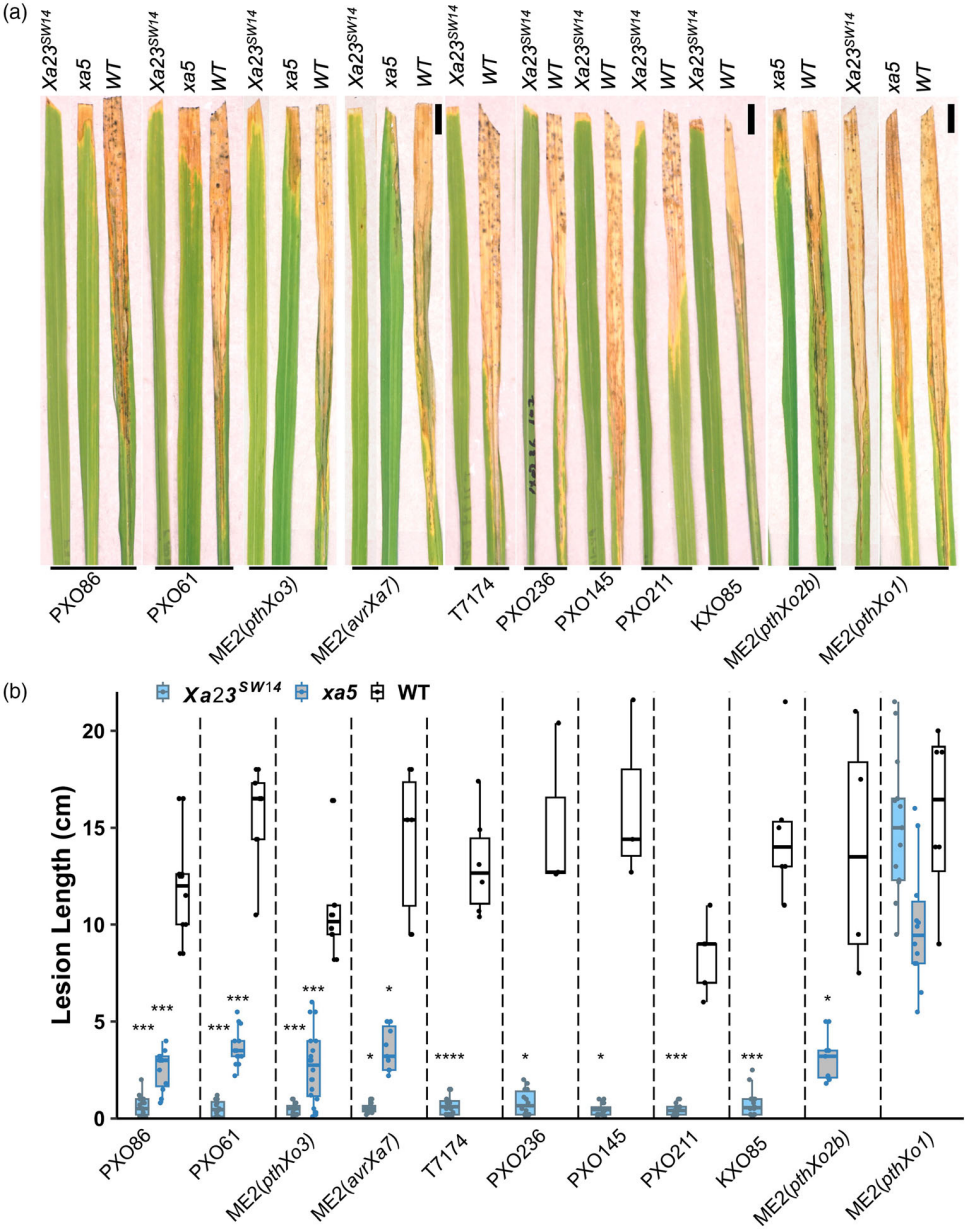
*Xa23* ^*SW14*^ and *xa5* alleles confer strong broad‐spectrum resistance to multiple pathogen strains. (a) Disease symptom (lesions) of edited homozygous T_1_ lines of *Xa23* ^*SW14*^ and *xa5*. Rice lines of different genotypes and *Xoo* strains used for inoculation are indicated above and below the leaf images, respectively. (b) Lesion lengths of rice leaves inoculated with different *Xoo* strains (as labelled below the graphs). Lesion lengths were measured 12 days postinoculation on at least three leaves of individual plants (*n* > 3). Scale bar, 1 cm. In bar graph, * represents *P*‐value <0.05, *** represents *P*‐value <0.001 and **** represents *P*‐value <0.0001 with *t*‐test.

All these homozygous T_1_ lines were challenged against several *Xoo* strains to test their expected broad‐spectrum resistance. For *Xa23* ^*SW14*^ edited lines, *Xoo* strains PXO86 (*avrXa7*), T7174 (*avrXa7*), PXO61 (*pthXo3* and *pthXo2*), PXO236 (*avrXa7* and *pthXo2*), PXO145 (*avrXa7*), PXO211 (*avrXa7*), KXO85 (*avrXa7*), ME2(*pthXo3*), ME2(*avrXa7*) were selected, while for *xa5* edited lines, PXO61 (*pthXo3* and *pthXo2*), PXO86 (*avrXa7*), ME2(*pthXo3*), ME2(*avrXa7*) and ME2(*pthXo2B*) were selected for leaf tip‐clipping inoculation. ME2(*pthXo1*) strain was used as control for both *Xa23* ^*SW14*^ and *xa5* lines. Lesion lengths were measured 12 days postinfection. The *Xa23* ^*SW14*^ edited plants were completely resistant against all the *avrXa7/pthXo3* harbouring Asian *Xoo* strains and the ME2 strains compared with the WT lines (Figure 3a,b). Similarly, the *xa5* edited plants were completely resistance to all the tested strains carrying three different TALEs (Figure 3a,b). This demonstrates the *Xa23* ^*SW14*^ and *xa5* alleles can render the otherwise susceptible rice cultivar with strong broad‐spectrum resistance. Both the edited lines were susceptible to ME2(*pthXo1*) which indicates that the resistance is specific to the edits and *pthXo1* is still virulent in *Xa23* ^*SW14*^ and *xa5* backgrounds. The T_1_ lines were visually monitored for general agronomic traits. No aberrant phenotype was observed in any of the edited lines compared with WT and the plant growth, development, reproductive transitioning, flowering, tillering and yield all were similar to those of WT line (data not collected). This suggests there are no visible pleiotropic effects associated with these two new alleles.

### Whole‐genome resequencing reveals no evidence of *OsMLH1dn*‐associated random mutations and PE off‐targeting in population

**Figure 4:**
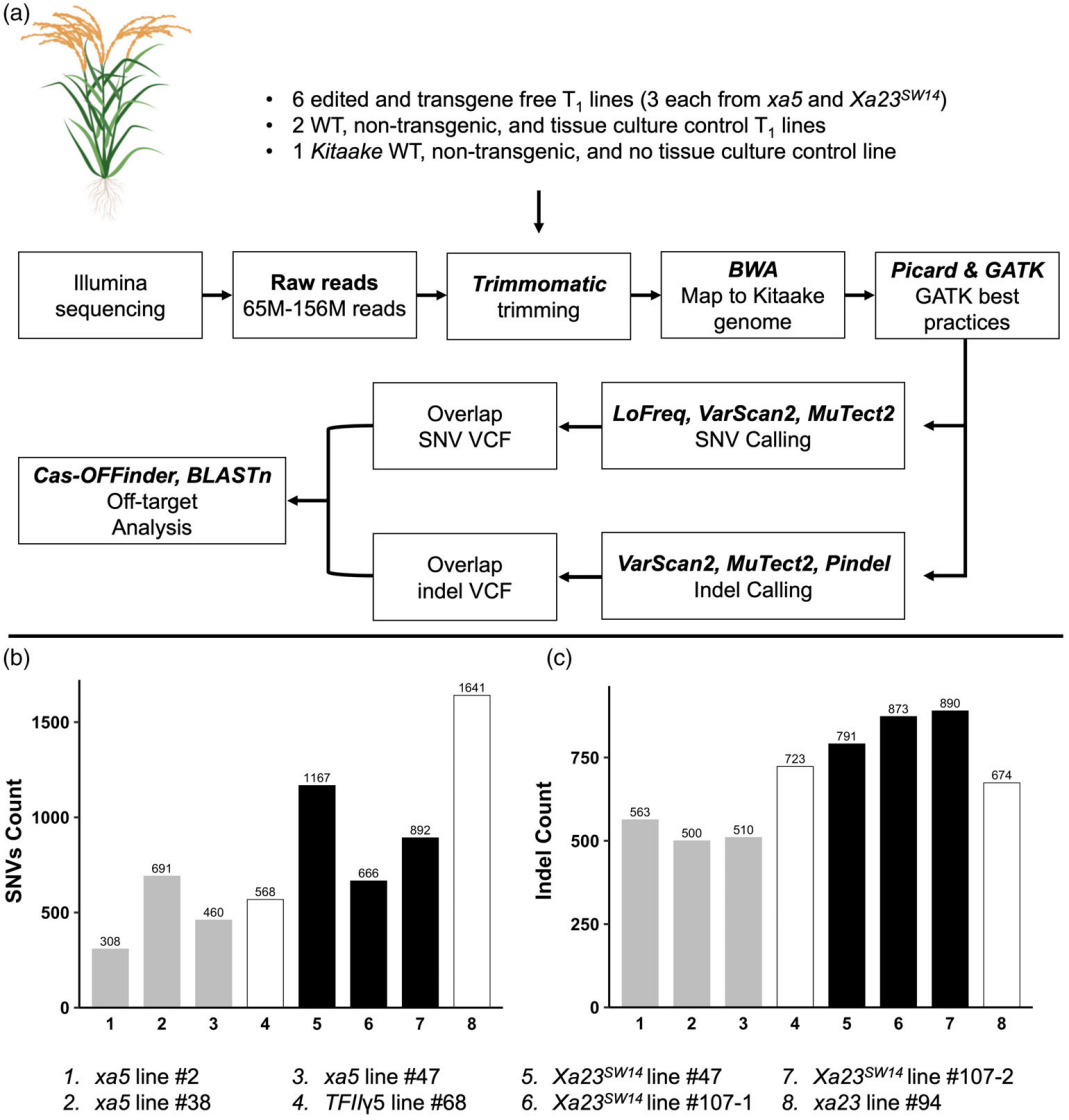
Whole‐genome sequencing revealed no off‐targets and *OsMLH1dn*‐associated variations. (a) The experimental design for the WGS and the flowchart of analysis pipeline. (b and c) Single Nucleotide Variations (SNVs) and indel counts from the final SNV and indel VCF files. The numbers 1–8 on *x*‐axis of both the bar graphs represent the plant lines that were sequenced, and the labels are mentioned in the bottom panel. The grey bars represent *xa5* edited lines, black bars correspond to *Xa23* ^*SW14*^ edited lines and the white bars indicate unedited wild‐type lines.

We whole‐genome sequenced nine rice lines to investigate the effect of OsMLH1dn, the negative dominant mismatch repair protein, on induction of random/spontaneous mutation as well as to quantify off‐target editing at the genome‐wide scale. We selected six edited but Cas9‐free plants (three each from the *xa5* and *Xa23* ^*SW14*^ backgrounds) as the test samples, two plants derived from T_0_ lines lacking PE constructs for editing of *xa5* and *Xa23* ^*SW14*^ (one from each PE construct) as the tissue culture control samples, and one Kitaake parent plant as a non‐tissue‐culture control for Illumina based sequencing at a minimum depth of 40X. Short (150‐bp) paired‐end raw sequences of 65–156 million reads from individual lines were obtained and processed for quality analysis, adapter trimming and low‐quality read filtering (Table S4). The remaining high‐quality reads were mapped to the Kitaake reference genome (Jain *et al*., 2019) and the variants were called using three different programmes for single nucleotide variations (SNVs) and indels against the Kitaake re‐sequenced line (Figure 4a). The overlapping SNVs and indels from three programmes were concluded to be true high‐quality variants and used for further off‐target analysis (Figure 4a). To investigate any mutations potentially caused by *OsMLH1dn*, we compared the number of SNVs and indels obtained in six edited lines which carried *OsMLH1dn* in T_0_ generation but segregated out to a non‐edited line that never carried the *OsMLH1dn* but went through tissue culture. The non‐*OsMLH1dn* carrying lines had equal or higher number of SNVs and indels compared with the lines which had *OsMLH1dn* in T_0_ generation (Figure 4b,c). This suggests that *OsMLH1dn* does not increase any spontaneous mutations in these transgenic plants. Furthermore, the edited lines do not share any common SNVs or indels among them or with the WT lines, implying that all the mutations are random, a characteristic feature of somatic mutations probably mainly induced by tissue culture process. This also suggests that there are no common off‐target edits in these edited lines.

To further investigate the off‐target editing scope in the PE lines, we searched for the top off‐target sites sharing substantial sequence homology with spacer sequences of pegRNAs. By allowing up to five mismatches, 12 and 13 potential off‐target sites for *xa5* and *Xa23* ^*SW14*^ spacer sequences were found, respectively, with both NGG and the non‐canonical NAG PAM sequences. All these potential off‐target sites were analysed in a 200‐bp window to detect any variant reads. While all the on‐target reads carry the desired edits, none of the potential off‐target sites carried any variant record in the overlap variant file. We further searched the raw variant files to detect any variants under lower stringency. No variants were found in any of these files in the potential off‐target sites or in the 200‐bp vicinity of these predicted off‐targets (Table 2a,b). These results demonstrate that the PE5max system has a very high on‐target efficiency and undetectable off‐targeting activity in our experiments.

**Table 2:** (a) Off‐target analysis in *Xa23* ^*SW14*^ edited lines. (b) Off‐target analysis in *xa5* edited lines

On/off‐target	Mismatch	Coordinates	# of Edited/WT reads			
			*Xa23* ^*SW14*^ line #47	*Xa23* ^*SW14*^ line #107‐1	*Xa23* ^*SW14*^ line #107‐2	*xa23* line #94
**GTAGCTGATGTTAGTGAGGCGG **	0	Chr11:22626881‐22626902	290/0	112/0	194/0	0/70
**GTAGCTGATGTatGTGAaGAGG **	3	Chr1:43907988‐43908010	0/108	0/74	0/56	0/31
**GgAtCTGATtTTAGTGAGGATG **	3	Chr2:34926017‐34926039	0/139	0/81	0/61	0/56
**GTAGCcGATcTTAGTaAGGTGG **	3	Chr2:14042273‐14042294	0/128	0/105	0/82	0/59
**GTcGCTGATGcTAGcGAGGAGG **	3	Chr11:22377056‐22377077	0/66	0/53	0/74	0/54
**GTAGCTGgTtTTgGTGAaGTGG **	3	Chr8:2676482‐2676503	0/128	0/73	0/74	0/52
**GgAGaTGATGTggGTGAGGAGG **	4	Chr8:20278075‐20278096	0/174	0/117	0/97	0/113
**GTAcCTGcTGTTAGTaAGa TGG **	4	Chr5:3854944‐3854965	0/119	0/59	0/72	0/63
**GTAGaTcATtTTAGTGAGc AGG **	4	Chr2:33367743‐33367764	0/128	0/109	0/105	0/108
**GTAGaTcATtTTAGTGAGc AGG **	4	Chr5:23200527‐23200548	0/124	0/89	0/105	0/104
**GcAGCTGATGTTgGTagGGCGG **	4	Chr1:154418‐154439	0/194	0/128	0/111	0/77
**GTtGaTGATGaTgGTGAGGAGG **	4	Chr1:2579928‐2579949	0/160	0/94	0/114	0/104
**GTAGaTGATGTTgtTGAGtGGG **	4	Chr1:7018780‐7018801	0/114	0/86	0/34	0/43
**GTAtCaGATtTTAGgGAGGAGG **	4	Chr1:8022585‐8022606	0/114	0/70	0/54	0/22

On/off‐target	Mismatch	Coordinates	# of Edited/WT reads			
			*xa5* line #2	*xa5* line #38	*xa5* line #47	*TFIIγ5* line #68
**AAGTAGATACCTTATCAAACTGG **	0	Chr5:595801‐595823	44/0	89/0	84/0	0/67
**AAGTAtATACtTTATtAAACTAG **	3	Chr1:39189985‐39190007	0/59	0/89	0/64	0/51
**AAGaAGATACCaTATCAtAa AAG **	3	Chr8:18790098‐18790120	0/37	0/82	0/55	0/55
**AAaTgGgTgCCTTATCAAACGGG **	4	Chr5:19793989‐19794011	0/56	0/101	0/54	0/45
**AtGTAcATtCaTTATCAAACTGG **	4	Chr5:20159282‐20159304	0/30	0/61	0/18	0/45
**AAGgAGcTACCTgATCcAACGGG **	4	Chr2:295806‐295828	0/48	0/93	0/59	0/46
** cAcTAGATAtaTTATCAAACAGG **	4	Chr2:36483137‐36483159	0/63	0/108	0/67	0/75
**AAGTAGATAaggTAgCAAACAGG **	4	Chr12:18787765‐18787787	0/57	0/92	0/67	0/58
** gAGaAGATACCaTATCAAAa GGG **	4	Chr9:11841117‐11841139	0/27	0/65	0/40	0/53
**AAGaAaATACCTTATCAAca TGG **	4	Chr10:10933734‐10933756	0/38	0/54	0/58	0/35
**AAGTAGAagCCgTAcCAAACGGG **	4	Chr10:11095538‐11095560	0/49	0/101	0/55	0/48
**AgGTttATACCTTATgAAACTGG **	5	Chr10:23990063‐23990085	0/63	0/101	0/79	0/63
**AAtTAGATcaCTgATCAAAa TGG **	5	Chr8:1070980‐1071002	0/67	0/80	0/46	0/26

## Discussion

Prime editing has been proved to be an efficient genetic tool, if highly efficient, for many applications, and even superior to CRISPR‐Cas nuclease‐based DNA modifications and base editing in some cases (Chen and Liu, 2022). One such application is for knock‐in of relatively large DNA fragments of various functionalities in endogenous context, for example cis‐regulatory elements (TALE EBEs in this case) and epitope‐tagging (FLAG tag, HiBiT luciferase, GFP11 or cellular localization signals) (Leonetti *et al*., 2016; Nelson *et al*., 2022; Schwinn *et al*., 2018). With the strategy to design pegRNA and ngRNA for knock‐in along with the highly efficient PE5max as demonstrated in this study, we anticipate the wide application of precise DNA insertions in plant and animal research.

Breeding for bacterial blight resistance has been the major interest of rice breeders. Novel strategies to create BB resistance are required to compete with the rapidly evolving *Xoo* populations by adding more layers of resistance. Precise genomic alterations using prime editing hold the greatest potential to generate novel genetic variations or germplasm in this regard. Here, we demonstrate such feasibility at high frequency by utilizing a high‐efficiency PE5max system to generate two novel resistant loci in highly susceptible rice Kitaake. In first instance, we edited the general transcription factor *TFIIAγ5* at amino acid position 39 from Valine to Glutamic acid (V39E) to recreate a recessive resistance gene *xa5*, and in second case, we engineered the dysfunctional *xa23* gene into dominant resistance genes *Xa23* ^*SW14*^ to carry EBE of *Xoo* virulence proteins AvrXa7 and PthXo3.

In both editing events, a high percentage of biallelically edited events was obtained (18% for *Xa23* ^*SW14*^ and 30.7% for *xa5*) out of the total lines screened. These numbers are extremely high as compared to any of the previous PE reports (Jiang *et al*., 2022; Lin *et al*., 2020, 2021; Zong *et al*., 2022) along with the original report of PE5max system in rice (Jiang *et al*., 2022). Although the use of high‐efficiency PE5max system accompanied with engineered pegRNA expression is responsible for major portion of this (Jiang *et al*., 2022), we also believe the use of high‐temperature condition of 30 °C in our tissue culture procedure might play a supplementary role in enhancing the nCas9‐RT activity as observed in an earlier report (Zou *et al*., 2022). This hypothesis needs further testing with multiple targets and different types of edits. For *xa5*, along with high frequency of edited lines we recovered a high portion of lines with desired edits (100% of the biallelic lines). We used the ngRNA to target the mutant strand for *xa5* editing. Initially, we hypothesized that nicking the edited strand would favour the edited flap over the WT flap during flap excision‐repair and enhance the efficiency, but with our results, it seems like this same strategy might be helpful in favouring the desired edit over the guide RNA scaffold derived by‐products. This hypothesis also needs to be further validated using more targets.

In this report, we demonstrated a high‐efficiency knock‐in of 30‐bp sequence using the PE5max system. None of the previous plant PE studies have tested knock‐in of DNA fragments of such size in transgenic plants, although larger fragment knock‐in is challenging using the current PE protocols due to the instability of large RNA reverse transcription template. But, the latest technologies such as GRAND editing (Wang *et al*., 2022) and TwinPE (Anzalone *et al*., 2022) can overcome these challenges when combined with PE5max system in plants. GRAND editing can efficiently instal 250‐bp DNA sequences with up to 27% conversion at endogenous loci and in non‐dividing mammalian cells (Anzalone *et al*., 2022; Wang *et al*., 2022), while TwinPE can insert 38‐bp attB or 50‐bp attP sequences at specified target DNA sites in mammalian cells with high efficiency, followed by Bxb1‐mediated integration of a 5.6‐kb plasmid donor at this landing pad site. This suggests that PE5max system has a very high scope of large fragment knock‐in in plants when co‐opted by GRAND and TwinPE editing strategies.

Although PE system has been reported to have lower off‐target editing as compared to double‐strand break repair mediated genome editing, higher editing efficiencies of PE5max system demand a thorough investigation of off‐target editing at a genome‐wide scale. Also, a genome‐wide sequence analysis is needed to capture the effect of OsMLH1dn, which is supposed to increase the PE editing efficiency. To carry this out, we sequenced six edited lines and compared them with the two WT lines to measure off‐targets at whole‐genome level. No evidence of off‐target or random mutations was found in any of the edited lines. Although there needs to be a further investigation of off‐targeting with more pegRNA targets, in this study, we can conclude that the PE5max system has very low, if not zero, off‐targeting.

The *xa5* allele in rice IRBB5 has been known to provide a broad‐spectrum resistance against all Asian *Xoo* strains except for the ones carrying *pthXo1* TALE virulence gene (Huang *et al*., 2016). *Xa23*(or *xa23*), if transcriptionally activated, is expected to become resistant against any *Xoo* strain carrying the cognate TALE gene (Wang *et al*., 2015). To test the effectiveness and specificity of these two alleles in the prime edited lines in Kitaake genetic background, we selected PXO86, an *avrXa7* harbouring strain, to challenge both *xa5* and *Xa23* ^*SW14*^ carrying lines and PXO99, a *pthXo1* carrying strain, to challenge *xa5* and *Xa23* ^*SW14*^ carrying lines, respectively, in T_0_ generation. Indeed, the resistance was effective against PXO86 but not against PXO99 in *xa5* lines. Similarly, *Xa23* ^*SW14*^ plants were resistant against PXO86. Further to test the broad‐spectrum scope of these two edits, we challenged the homozygous T_1_ lines of both *xa5* and *Xa23* ^*SW14*^ against multiple *Xoo* strains. All the lines became completely resistant against all the tested *Xoo* strains except for the ones carrying the TALE *pthXo1*. This indicates that both the alleles are effective in providing broad‐spectrum resistance against multiple *Xoo* strains and the resistance is specific to the edits. This is the first‐ever study to utilize *xa5* allele in any rice line other than the rice cultivar IRBB5 to provide resistance and the first study to validate the role of V39E substitution of *TFIIAγ5* in providing resistance in different genetic background.

*xa5* is a rare allele to confer broad‐spectrum and durable resistance against BB in rice (Huang *et al*., 2016). We searched the 3017 rice accessions for *xa5* allele using the SNP‐seek database (Mansueto *et al*., 2017). Out of 3017 rice accessions, only 95 carry the *xa5* allele which is 3.14% of the total. Breeding of this allele using conventional approaches could take years and can be hindered by unwanted traits through linkage drag and crossing barrier between *japonica* and *indica* cultivars (Zhu *et al*., 2017). Furthermore, the available executor *R* genes in nature do not carry all the EBEs of known TALE proteins. Although the utility of promoter trap strategy has been demonstrated (Wei *et al*., 2021), prime editing offers advantages such as higher editing efficiency and lower or no Cas9/donor template‐mediated off‐targeting. Also, the *Xa23* ^*SW14*^ is a novel allele in such a way that it utilizes the vulnerable host element from the S gene *SWEET14* to trap the AvrXa7 and PthXo3 TALEs. The same strategy can be used to exploit other BB susceptibility gene EBEs for resistance against the cognate virulent TALEs. *Xa23* ^*SW14*^ has the potential to provide more durable resistance compared with original *Xa23* from IRBB23 rice whose resistance can be broken by mutations in TALE repeat units due to no penalty in fitness even upon loss of *avrXa23*. Prime editing, therefore, offers the advantage to breed these alleles in elite cultivars in a single generation and a few rounds of back‐crossings to obtain pure background and be free of transgene. Although this study was conducted on the model rice Kitaake to test the efficacy of these alleles against *Xoo*, similar outcomes can be expected from elite lines with these alleles. Similarly, EBE‐engineered executor *R* genes offer a great potential to fight off the rapidly evolving *Xoo* strains. We searched the rice genome for executor‐like *R* genes and found several such dysfunctional executor genes. The activity of these executor‐like genes needs to be tested using designer TALEs or using the EBE engineering approach. However, these could potentially be utilized as a weapon against multiple *Xoo* strains by stacking multiple EBEs into the promoter of a single executor gene or individual EBEs into the promoters of multiple executor genes, a strategy like *do novo* evolution.

## Materials and methods

All the primers used in this study are listed in Table S1, and the pegRNAs or ngRNAs are listed in Table S2.

### Plant materials, bacterial strains, medium and growth conditions

The *japonica* rice cultivar Kitaake was used for all the editing experiments. *Xoo* strains were from the collection of the Yang laboratory.

All rice plants were grown in the greenhouse and growth chambers with a 12‐h/30 °C light period and a 12‐h/28 °C dark period at 60%–75% relative humidity. *Escherichia coli* and *Agrobacterium tumefaciens* strains were grown in Luria‐Bertani medium supplemented with appropriate antibiotics at 37 and 28 °C, respectively. *Xoo* was grown at 28 °C on TSA (10 g/L tryptone, 10 g/L sucrose, 1 g/L glutamic acid and 1.5% Difco agar). Antibiotics were used at the following concentrations if required: 25 μg/mL rifampicin, 50 μg/mL kanamycin and 100 μg/mL spectinomycin.

### Disease assays

The leaf tip‐clipping method was used to assess the disease phenotypes of edited rice as described previously (Yang and Bogdanove, 2013). In brief, an aliquot of the appropriate *Xoo* glycerol stock, stored at −80 °C, was streaked onto TSA containing appropriate antibiotics and grown at 28 °C for about 3 days. The bacterial cells were harvested from plates, suspended in sterile water, washed twice with water and resuspended in water; the solution was adjusted to an optical density of 0.5 at 600 nm. Scissor blades were immersed in the *Xoo* suspension and used to clip the tip of a fully expanded leaf. The lesion lengths were measured at 12 days or at the specified days after inoculation. Three to five replicates with multiple leaves per replicate were examined for each *Xoo* strain. Data were plotted using ggplot2 (Villanueva and Chen, 2019) and ggpubr (Kassambara and Kassambara, 2020) package from R software. Two‐tailed Student's *t*‐test with or without Bonferroni correction for multiple comparisons was conducted using the R package rstatix (Kassambara, 2020), and Tukey's *post hoc* tests were conducted using R package agricolae (de Mendiburu and de Mendiburu, 2019).

### PE plasmid construction and rice transformation

The PE5max system was used for this study (Jiang *et al*., 2022). The pegRNA and ngRNA were cloned using the overlap PCR‐based approach as described previously (Jiang *et al*., 2022) (Figure S5). Rice cultivar Kitaake (*Oryza sativa* spp. *japonica*) was used for *Agrobacterium‐*mediated transformation using a slightly modified protocol as described (Hiei *et al*., 1994). Briefly, embryos from mature seeds were used for callus induction in Murashige and Skoog (MS) medium supplemented with 2 mg/L of 2,4‐dichlorophenoxyacetic acid (2,4‐D). Callus cells derived from scutella were co‐cultivated with *Agrobacterium* strain LBA4404/pVS1‐VIR2 carrying the appropriate PE plasmids. Inoculated callus cells were cultured in MS medium supplemented with 2,4‐D (2 mg/L), hygromycin (50 mg/L) and Timentin (200 mg/L) for hygromycin‐resistant callus lines for two rounds of selection (14 days per round). Hygromycin‐resistant callus lines were transferred to regeneration medium (MS supplemented with BAP and NAA) to regenerate embryogenic shoots. The shoots were transferred into rooting medium (½ MS medium supplemented with 25 mg/L hygromycin) to generate roots before being transferred into soil and grown in greenhouse.

### RNA isolation and gene expression analysis

Total RNA was isolated from the *Xoo* syringe infiltrated leaves of PE edited and WT Kitaake lines followed by Dnase (Thermo Fisher Scientific Inc.) treatment to remove DNA. The RNA quality was assessed using agarose gel and concentration was measured on NanoDrop (Thermo Fisher Scientific Inc.). A total of 1 μg RNA were used for first‐strand cDNA synthesis using the iScript cDNA Synthesis Kit (Bio‐Rad). The cDNA was diluted 1 : 20 times and used directly for PCR using gene‐specific primers.

### Genotyping of PE callus lines and plants of T_0_ and T_1_ generations and deep sequence analysis

DNA from the T_0_ and T_1_ lines was isolated using the CTAB method. Primers flanking the target sites were used to PCR‐amplify the target regions following digestion with appropriate enzymes to detect editing events. The PCR amplicons of edited lines were deep‐sequenced using the Illumina MiSeq PE150. Briefly, the 150–250 bp region flanking the target site was amplified using gene‐specific primers extended by sequencing primers in the first round of PCR. In the second nested PCR, the dual barcoded Illumina adapters were used to make the gene‐specific products from the first round. The PCR products were purified using columns, pooled in equal amounts, and subjected to sequencing in the DNA sequencing core facility at the University of Missouri‐Columbia. Demultiplexed and trimmed reads were obtained from the facility. The analysis was performed using CRISPResso2 with the default setting for both NHEJ and prime‐editing output (Pinello *et al*., 2016).

### WGS and data analysis

DNA was extracted from single seedlings using CTAB method, followed by RNase treatment (Thermo Fisher Scientific Inc.) and column purification. Quality of DNA was assessed using agarose gel and NanoDrop (Thermo Fisher Scientific Inc.). All samples were sequenced by Novogene using Illumina paired‐end technology. Raw reads of size 2× 150‐bp were obtained from the company and processed for WGS analysis following a previously described protocol (Liu *et al*., 2021). Briefly, raw reads were assessed for quality using FastQC (Andrews, 2010) and multiQC (Ewels *et al*., 2016). Adapters and low‐quality reads were trimmed using trimmomtaic (Bolger *et al*., 2014) with parameters LEADING:3 TRAILING:3 MINLEN:36. Trimmed reads were aligned to Kitaake reference genome (Jain *et al*., 2019) using BWA (Li and Durbin, 2009). The aligned sam files were sorted for coordinates using picard SortSam (https://github.com/broadinstitute/picard) and converted to bam files and indexed using samtools (Li *et al*., 2009). The reads near indels were realigned and base quality scores were recalibrated using GATK best practices (McKenna *et al*., 2010). The resulting bam files were called for single nucleotide variations (SNVs) using three software; LoFreq (Wilm *et al*., 2012), MuTect2 (McKenna *et al*., 2010) and VarScan2 (Koboldt *et al*., 2012). Indels were also called using three software: MuTect2 (McKenna *et al*., 2010), VarScan2 (Koboldt *et al*., 2012) and Pindel (Ye *et al*., 2009). Overlapping SNVs/indels were obtained using bedtools (Quinlan and Hall, 2010) and BCFtools (Danecek *et al*., 2021). Cas‐OFFinder online tool (Bae *et al*., 2014) and BLASTn with ‐task blastn‐short (Altschul *et al*., 1990) were used to predict off‐target sites by allowing up to 5‐nt mismatch. Samtools (Li *et al*., 2009) was used to count reads around on/off‐target sites. Data were analysed using R and Python, and graphs were made in R.

### Statistics and data analysis

Data were analysed and plotted using R packages ggplot2 (Villanueva and Chen, 2019), ggpubr (Kassambara and Kassambara, 2020), rstatix (Kassambara, 2020) and agricolae (de Mendiburu and de Mendiburu, 2019). For Figures 1f, 2f and Figure S4b, two‐tailed *t*‐test adjusted using Bonferroni correction for multiple comparisons was used. For Figure 3b,d, two‐tailed *t*‐test was used.

## Competing interests

The authors declare no competing interests.

## Author contributions

A.G. and B.Y. designed the research and wrote the paper with the revision from other authors. A.G and B.L. performed the research. Q.C., A.G. and B.Y. analysed the data.

## Supporting information


**Table S1** Primer sequences.
**Table S2** Sequences related to pegRNAs and ngRNAs.
**Table S3** Genotyping and deep‐sequecing of *xa23/Xa23SW14* lines.
**Table S4** Whole genome Illumina re‐seqeuencing sample information.
**Figure S1** Schematic representation of *xa5* mediated recessive and Executor *R* gene mediated dominant resistance mechanisms in rice.
**Figure S2** Promoter regions of *Xa23* (*xa23*) alleles for editing.
**Figure S3** Induction of *Xa23SW14* by *avrXa7*‐carrying PXO86.
**Figure S4** Disease assay on *TFIIAγ5/xa5* edited biallelic, monoallelic, and WT lines.
**Figure S5** PE5max construct used for *Agrobacterium* mediated rice transformation.

## Data Availability

The WGS data are available on NCBI under BioProject accession PRJNA916353 from SRR22904513 to SRR22904521 accession numbers. The plant materials and constructs generated in this study are available upon request.
