# Supplementary material for: High‐efficiency prime editing enables new strategies for broad‐spectrum resistance to bacterial blight of rice
Source: Plant Biotechnol J. 2023 May 3;21(7):1454–64. doi: 10.1111/pbi.14049 (PMC10281596; doi:10.1111/pbi.14049)
Supplement: Supplementary file 1 — Table S1 Primer sequences. Table S2 Sequences related to pegRNAs and ngRNAs. Table S3 Genotyping and deep‐sequecing of xa23/Xa23SW14 lines. Table S4 Whole genome Illumina re‐seqeuencing sample information. Figure S1 Schematic representation of xa5 mediated recessive and Executor R gene mediated dominant resistance mechanisms in rice. Figure S2 Promoter regions of Xa23 (xa23) alleles for editing. Figure S3 Induction of Xa23SW14 by avrXa7‐carrying PXO86. Figure S4 Disease assay on TFIIAγ5/xa5 edited biallelic, monoallelic, and WT lines. Figure S5 PE5max construct used for Agrobacterium mediated rice transformation. [file PBI-21-1454-s001.pdf]

Supplementary information

High-efficiency prime editing enables new strategies for broad-spectrum resistance to bacterial blight of rice

Ajay Gupta[1], Bo Liu[1], Qi-Jun Chen[2,3], Bing Yang[1,4,*]

[1] Division of Plant Science and Technology, Bond Life Sciences Center, University of Missouri, Columbia, MO 65211, USA
[2] State Key Laboratory of Plant Physiology and Biochemistry, College of Biological Sciences, China Agricultural University, Beijing 100193, China
[3] Center for Crop Functional Genomics and Molecular Breeding, China Agricultural University, Beijing 100193, China
[4] Donald Danforth Plant Science Center, St. Louis, MO 63132, USA

* Corresponding authors
Bing Yang (yangbi@missouri.edu)

Supplementary Tables

Suppl. Table 1: Primer sequences.
Suppl. Table 2: Sequences related to pegRNAs and ngRNAs.
Suppl. Table 3: Genotyping and deep-sequecing of *xa23/Xa23*^{SW14} lines.
Suppl. Table 4: Whole genome Illumina re-seqeuencing sample information

Supplementary Figures

Suppl. Fig.1. Schematic representation of *xa5* mediated recessive and Executor *R* gene mediated dominant resistance mechanisms in rice.
Suppl. Fig 2. Promoter regions of *Xa23* (*xa23*) alleles for editing.
Suppl. Fig. 3. Induction of *Xa23*^{SW14} by *avrXa7*-carrying PXO86.
Suppl. Fig. 4. Disease assay on *TFIIAγ5/xa5* edited biallelic, monoallelic, and WT lines.
Suppl. Fig. 5. PE5max construct used for *Agrobacterium* mediated rice transformation.

Supplementary Table 1: Primer Sequences

Name	Sequence (5´ - 3´)	Purpose
iSce-R1	AACCACCATTACCCTGTTATCCCTA	PE plasmid construction
BsaOL-R	ACATTATTTGGTCTCTAAAC	
gXa5gRm-F1	ATTATTATTGGTCTCATGCAaagtagataccttatcaaac	PE plasmid construction for *xa5* editing
gXa5gRm-F1b	aagtagataccttatcaaacGTTTAAGAGCTATGCTGGAA	
gXa5RP-F2	ATTATTATTGGTCTCAGTGCGCCATTCAAGTTCTTGagCAGTT	
gXa5RP-F2b	CAAGTTCTTGagCAGTTTGATAAGGTATCAGGAATAA	
gXa5evo-F	TGATAAGGTATCAGGAATAACGCGGTTCTATCTAGTTAC	
gXa5tMet-R1	ACATTATTTGGTCTCTAAACTATCAAACTGctCAAGAACT	
gXa5tMet-R2	ACTATCAAACTGctCAAGAACTTATCAGAGCCAGGTTTC	
gXa23gRm-F1	ATTATTATTGGTCTCATGCAgtagctgatgttagtgagg	PE plasmid construction for AvrXa7 EBE knock-in in *xa23*
gXa23gRm-F1b	AgtagctgatgttagtgaggGTTTAAGAGCTATGCTGGAA	
gXa23RP-F3	ATTATTATTGGTCTCAGTGCGCCTTCCTTCCGCCTtatataaaccccct	
gXa23RP-F3b	CTTCCGCCTtatataaaccccctccaaccaggtgctaagCACTAACATCAGCAAATTCAT	
gXa23evo-F	CACTAACATCAGCAAATTCATCGCGGTTCTATCTAGTTAC	
gXa23tMet-R3	ACATTATTTGGTCTCTAAACtgagatgatgcaacaaggaac	
gXa23tMet-R3b	ACtgagatgatgcaacaaggaacTATCAGAGCCAGGTTTCG	
yCLV-F1	TAGCCTAGAAGTAGTCAAGG	Screening and sequencing of PE plasmids
pegHSP-R1	CAATTAGCCCCGGAGATATC	
Ga5RT-F	CTCCGCTCCTCCTCTCCTTGC	Amplifying *xa5* flanking PE target site
Xa5-R1	gctgaatccacaaccaagtg	
xa23p-F1	CCCGAACATCACTAACATCG	Amplifying *xa23* flanking PE target site
xa23HR-R8	TCATGTGTATACCGGCTACGG	
xa23RT-F2	CCTGTTTGGCTCCATCATGC	RT-PCR of *xa23*
xa23RT-R1	GGGAGAATAACCATCTTGTCGTC	
OsActinF3	ctcagcacattccagcagat	RT-PCR of *Actin*
OsActinR3	ACAGATAGGCCGGTTGAAAA	
xa23p-F3	CTCTTTCCCTACACGACgctcttccgatctTCGTCGTGGTGGTGAGATTAAG	Deep amplicon sequencing of *xa23* PE edits
Xa23HR-R11	ctggagttcagacgtgtgctcttccgatctTCATGTGTATACCGGCTACGG	
Xa5-F1	CTCTTTCCCTACACGACgctcttccgatctCATTGGCATGTGCCTCACTG	Deep amplicon sequencing of *TFIIAγ5* PE edits
Xa5-R2	ctggagttcagacgtgtgctcttccgatctCTCCAAGGCTTCCGTCATAG	
Truseq-R49	CAAGCAGAAGACGGCATACGAGAT**CGAGTAAT**GTGACTGGAGTTCAGACGTGTGCT	Deep amplicon sequencing with dual
Truseq-R50	CAAGCAGAAGACGGCATACGAGAT**TCTCCGGA**GTGACTGGAGTTCAGACGTGTGCT	

Truseq-R51	CAAGCAGAAGACGGCATACGAGAT**AATGAGCG**GTGACTGGAGTTCAGACGTGTGCT	multiplexed barcodes (bold and underlined)
Truseq-R52	CAAGCAGAAGACGGCATACGAGAT**GGAATCTC**GTGACTGGAGTTCAGACGTGTGCT	
Truseq-R53	CAAGCAGAAGACGGCATACGAGAT**TTCTGAAT**GTGACTGGAGTTCAGACGTGTGCT	
Truseq-R54	CAAGCAGAAGACGGCATACGAGAT**ACGAATTC**GTGACTGGAGTTCAGACGTGTGCT	
Truseq-R55	CAAGCAGAAGACGGCATACGAGAT**AGCTTCAG**GTGACTGGAGTTCAGACGTGTGCT	
Truseq-R56	CAAGCAGAAGACGGCATACGAGAT**GCGCATTA**GTGACTGGAGTTCAGACGTGTGCT	
Truseq-R57	CAAGCAGAAGACGGCATACGAGAT**CATAGCCG**GTGACTGGAGTTCAGACGTGTGCT	
Truseq-R58	CAAGCAGAAGACGGCATACGAGAT**TTCGCGGA**GTGACTGGAGTTCAGACGTGTGCT	
Truseq-R59	CAAGCAGAAGACGGCATACGAGAT**GCGCGAGA**GTGACTGGAGTTCAGACGTGTGCT	
Truseq-R60	CAAGCAGAAGACGGCATACGAGAT**CTATCGCT**GTGACTGGAGTTCAGACGTGTGCT	
TruSeq-F1	AATGATACGGCGACCACCGAGATCTACAC**TATAGCCT**ACACTCTTTCCCTACACGACGCTCT	
TruSeq-F2	AATGATACGGCGACCACCGAGATCTACAC**ATAGAGGC**ACACTCTTTCCCTACACGACGCTCT	
TruSeq-F3	AATGATACGGCGACCACCGAGATCTACAC**CCTATCCT**ACACTCTTTCCCTACACGACGCTCT	
TruSeq-F4	AATGATACGGCGACCACCGAGATCTACAC**GGCTCTGA**ACACTCTTTCCCTACACGACGCTCT	
TruSeq-F5	AATGATACGGCGACCACCGAGATCTACAC**AGGCGAAG**ACACTCTTTCCCTACACGACGCTCT	
TruSeq-F6	AATGATACGGCGACCACCGAGATCTACAC**TAATCTTA**ACACTCTTTCCCTACACGACGCTCT	
TruSeq-F7	AATGATACGGCGACCACCGAGATCTACAC**CAGGACGT**ACACTCTTTCCCTACACGACGCTCT	
TruSeq-F8	AATGATACGGCGACCACCGAGATCTACAC**GTACTGAC**ACACTCTTTCCCTACACGACGCTCT	

Supplementary Table 2: Sequences related to pegRNAs and ngRNAs

Gene	Edit	pegRNA/ngRNA	Sequence (5´ – 3´)
TFIIAγ5	V39E, TC>AG	target	AAGTAGATACCTTATCAAAC
		rtT/PBS/linker	GCCATTCAAGTTCTTGAGCAGTT/TGATAAGGTATC/AGGAATAA
		nicking gRNA	TATCAAACTGCTCAAGAACT
xa23	AvrXa7 EBE knock-in	target	GTAGCTGATGTTAGTGAGG
		rtT/PBS/linker	GCCTTCCTTCCGCCTTATATAAACCCCCTCCAACCAGGTGCTAAG/CACTAACATCA/AAATTCAT
		nicking gRNA	GTTCCTTGTTGCATCATCTCA

Supplementary Table 3: Genotyping and deep-sequecing of *xa23*/*Xa23*^{SW14} lines

Line	Edited sequence (5´ – 3´)	Edit type	# of reads	% of reads*	M/ B
#2	TCGTCGTGGTGGTGAGATTAAGCCTTCCTTCCGCCTTATATAAACCCCCTCCAACCAGGTGCTAAGCACT	Intact	1008	19.09%	M
	AGCCTTCCTTCCGCCTCACTAACATCAGCTACTATAAAAGCCCTTCCTTGTTGCATCATCTACTAACATC	Indel	1732	32.80%	
#4	TCGTCGTGGTGGTGAGATTAAGCCTTCCTTCCGCCTCACTATAAACCCCCTCCAACCAGGTGCTAAGCAC	Intact	1770	21.17%	M
	AGCCTTCCTTCCGCCTCACTAACATCAGCTACTATAAAAGCCCTTCCTTGTTGCATCAGCTACTATAAAA	Indel	4173	49.92%	
	AGATTAAGCCTTCCTTCCGCCT----AACATCAGCTACTATAAAAGCCCTTCCTTGTTGCATCATCTCAA	Indel	1187	14.20%	
#7	TCGTCGTGGTGGTGAGATTAAGCCTTCCTTCCGCCTTATATAAACCCCCTCCAACCAGGTGCTAAGCACT	Intact	2614	24.32%	M
#9	TCGTCGTGGTGGTGAGATTAAGCCTTCCTTCCGCCTTATATAAACCCCCTCCAACCAGGTGCTAAGCACT	Intact	3673	35.68%	M
#11	TCGTCGTGGTGGTGAGATTAAGCCTTCCTTCCGCCTTATATAAACCCCCTCCAACCAGGTGCTAAGCACT	Intact	12840	96.83%	B
#12	TCGTCGTGGTGGTGAGATTAAGCCTTCCTTCCGCCTTATATAAACCCCCTCCAACCAGGTGCTAAGCACT	Intact	7749	97.23%	B
#12a	TCGTCGTGGTGGTGAGATTAAGCCTTCCTTCCGCCTTATATAAACCCCCTCCAACCAGGTGCTAAGCACT	Intact	922	20.65%	M
#13	ATTAAGCCTACCAGGTGCTAAGCACTAACATCAGCTACTATAAAAGCCCTTCCTTGTTGCATCATCTCA	Partial	600	11.92%	M
	TCGTCGTGGTGGTGAGATTAAGCCTTCCTTCCGCCTTATATAAACCCCCTCCAACCAGGTGCTAAGCACT	Intact	826	16.41%	
	TCGTCGTGGTGGTGAGATTAAGCCTTCCTTCCGCCTCCTTCCGCCTTATATAAACCCCCTCCAACCAGGT	Intact	638	12.67%	
#16	TCGTCGTGGTGGTGAGATTAAGCCTTCCTTCCGCCTTATATAAACCCCCTCCAACCAGGTGCTAAGCACT	Intact	3208	25.23%	M
	ATTAAGCCTTCCAGGTGCTAAGCACTAACATCAGCTACTATAAAAGCCCTTCCTTGTTGCATCATCTCAA	Partial	3006	23.64%	
#17	AGATTAAGCCTTCA------------------AGC-ACTATAAAAGCCCTTCCTTGTTGCATCATCTCAA	Indel	5825	55.86%	M
	AGATTAAGCCTTCCTTCCGCCT---------------CAACCAGGTGCTAAGCACT	Partial	4602	44.14%	
#18	TCGTCGTGGTGGTGAGATTAAGCCTTCCTTCCGCCTTATATAAACCCCCTCCAACCAGGTGCTAAGCACT	Intact	8682	75.97%	B
#18a	TCGTCGTGGTGGTGAGATTAAGCCTTCCTTCCGCCTTATATAAACCCCCTCCAACCAGGTGCTAAGCACT	Intact	1751	34.85%	M
#19	TCGTCGTGGTGGTGAGATTAAGCCTTCCTTCCGCCTTATATAAACCCCCTCCAACCAGGTGCTAAGCACT	Intact	8878	65.53%	M
#22	TCGTCGTGGTGGTGAGATTAAGCCTTCCTTCCGCCTTATATAAACCCCCTCCAACCAGGTGCTAAGCACT	Intact	2983	14.94%	M
	ATTAAGCCTTCCAGGTGCTAAGCACTAACATCAGCTACTATAAAAGCCCTTCCTTGTTGCATCATCTCAA	Partial	2185	10.94%	
#25	TCGTCGTGGTGGTGAGATTAAGCCTTCCTTCCGCCTTATATAAACCCCCTCCAACCAGGTGCTAAGCACT	Intact	1766	11.82%	M
#26	TCGTCGTGGTGGTGAGATTAAGCCTTCCTTCCGCCTTATATAAACCCCCTCCAACCAGGTGCTAAGCACT	Intact	7416	46.61%	M
#26a	TCGTCGTGGTGGTGAGATTAAGCCTTCCTTCCGCCTTATATAAACCCCCTCCAACCAGGTGCTAAGCACT	Intact	2681	30.23%	M
	ATTAAGCCTTCCAGGTGCTAAGCACTAACATCAGCTACTATAAAAGCCCTTCCTTGTTGCATCATCTCAA	Partial	889	10.02%	

#27	GTCGTGGTGGTGAGATTAAGCCTTCCTTCCCCGCCTTATATAAACCCCCTCCAACCAGGTGCTAAGCACT	Intact	2323	25.28%	M
#27a	AGCCTTCCTTCCGCCTCACTAACATCAGCTACTATAAAAGCCCTTCCTTGTTGCATCATATAAAAGCCCT	Indel	1513	16.79%	M
	TCGTCGTGGTGGTGAGATTAAGCCTTCCTTCCGCCTTATATAAACCCCCTCCAACCAGGTGCTAAGCACT	Intact	1726	19.16%	
#29	TCGTCGTGGTGGTGAGATTAAGCCTTCCTTCCGCCTTATATAAACCCCCTCCAACCAGGTGCTAAGCACT	Intact	825	18.57%	M
	AGCCTTCCTTCCGCCTCACTAACATCAGCTACTATAAAAGCCCTTCCTTGTTGCATCATATAAAAGCCCT	Indel	1308	29.45%	
#30	TCGTCGTGGTGGTGAGATTAAGCCTTCCTTCCGCCTTATATAAACCCCCTCCAACCAGGTGCTAAGCACT	Intact	1825	25.58%	M
	ATTAAGCCTTCCAGGTGCTAAGCACTAACATCAGCTACTATAAAAGCCCTTCCTTGTTGCATCATCTCAA	Partial	713	9.99%	
#32	TCGTCGTGGTGGTGAGATTAAGCCTTCCTTCCGCCTTATATAAACCCCCTCCAACCAGGTGCTAAGCACT	Intact	1220	13.86%	M
	TGCCTTCCTTCCGCCTCACTAACATCAGCTACTATAAAAGCCCTTCCTTGTTACATCAGCTACTATAAAA	Indel	3210	36.47%	
#33	ATTAAGCCTTCCAGGTGCTAAGCACTAACATCAGCTACTATAAAAGCCCTTCCTTGTTGCATCATCTCAA	Partial	721	8.26%	M
	TCGTCGTGGTGGTGAGATTAAGCCTTCCTTCCGCCTTATATAAACCCCCTCCAACCAGGTGCTAAGCACT	Intact	997	11.42%	
#35	ACTAACATCAGCTACTATAAAAGCCCTTCCTTGTTGCATCATCTAAACCCCCTTCCAACCAGGTGCTAAG	Partial	9586	67.64%	M
#36	GATTAAGCCTTCCTTCCGCCTCACTAACATCAGCTACTATACCCCCTCCAACCAGGTGCTAAGCACT	Intact	8648	67.88%	M
#39	TCGTCGTGGTGGTGAGATTAAGCCT----------------AAACCCCCTCCAACCAGGTGCTAAGCACT	Partial	13865	94.62%	B
#40	TCGTCGTGGTGGTGAGATTAAGCCTTCCTTCCGCCTTATATAAACCCCCTCCAACCAGGTGCTAAGCACT	Intact	11604	98.64%	B
#41	TCGTCGTGGTGGTGAGATTAAGCCTTCCTTCCGCCTTATATAAACCCCCTCCAACCAGGTGCTAAGCACT	Intact	1814	18.42%	M
#45	CTTATATAAACCCCCTCCAACCAGGTGCTAAGCACTAACATCAGCTACTATAAAAGCCCTTCCTTGTTGA CATCAGCTACTATAAA	Intact	1454	59.25%	M
#47	ATTAAGCCTTCCAGGTGCTAAGCACTAACATCAGCTACTATAAAAGCCCTTCCTCAGCTACTATAAAAGC	Partial	4782	84.89%	B
#49	ATTAAGCCTTCCAGGTGCTAAGCACTAACATCAGCTACTATAAAAGCCCTTCCTCAGCTACTATAAAAGC	Partial	7411	98.47%	B
#56	TCGTCGTGGTGGTGAGATTAAGCCTTCCTTCCGCCTTATATAAACCCCCTCCAACCAGGTGCTAAGCACT	Intact	4414	51.30%	M
#60	CTTATATAAACCCCCTCCAACCAGGTGCTAAGCACTCACTAACATCAGCTACTATAAAAGCCCTTCCTTG TTGCATCATCTACTATAAAA	Intact	5536	96.60%	B
#61	TCGTCGTGGTGGTGAGATTAAGCCTTCCTTCCGCCTTATATAAACCCCCTCCAACCAGGTGCTAAGCACT	Intact	1172	15.59%	B
	TCGTCGTGGTGGTGAGATTAAGCCT-----------TATATAAACCCCCTCCAACCAGGTGCTAAGCACT	Intact	3479	46.28%	
#66	TCGTCGTGGTGGTGAGATTAAGCCTTCCTTCCGCCTTATATAAACCCCCTCCAACCAGGTGCTAAGCACT	Intact	3274	26.91%	M
#70	TCGTCGTGGTGGTGAGATTAAGCCTTCCTTCCGCCTCACTAACATCAACCCCCTCCAACCAGGTGCTAAG	Partial	1988	57.57%	M
#71	TCGTCGTGGTGGTGAGATTAAGCCCTTCCTTCCGCCTTATATAAACCCCCTCCAACCAGGTGCTAAGCAC T	Intact	3382	46.15%	M

#72	TCGTCGTGGTGGTGAGATTAAGCCCTTCCTTCCGCCTTATATAAACCCCCTCCAACCAGGTGCTAAGCAC T	Intact	8942	82.22%	B
#76	TCGTCGTGGTGGTGAGATTAAGCCTTCCTTCCGCCTTATATAAACCCCCTCCAACCAGGTGCTAAGCACT	Intact	9089	91.99%	B
#77	TCGTCGTGGTGGTGAGATTAAGCCTTCCTTCCGCCTTATATAAACCCCCTCCAACCAGGTGCTAAGCACT	Intact	3769	69.68%	M
#78	TCGTCGTGGTGGTGAGATTAAGCCTTCCTTCCGCCTTATATAAACCCCCTCCAACCAGGTGCTAAGCACT	Intact	10636	95.65%	B
#79	TCGTCGTGGTGGTGAGATTAAGCCTTCCTTCCGCCTTATATAAACCCCCTCCAACCAGGTGCTAAGCACT	Intact	1915	11.65%	M
	ATTAAGCCTTCCAGGTGCTAAGCACTAACATCAGCTACTATAAAAGCCCTTCCTTGTTGCATCATCTCAA	Partial	2000	12.16%	
#81	TCGTCGTGGTGGTGAGATTAAGCCTTCCTTCCGCCTTATATAAACCCCCTCCAACCAGGTGCTAAGCACT	Intact	1439	14.94%	M
#84	TCGTCGTGGTGGTGAGATTAAGCCTTCCTTCCGCCTTATATAAACCCCCTCCAACCAGGTGCTAAGCACT	Intact	2047	21.17%	M
	AGCCTTCCTTCCGCCTCACTAACATCAGCTACTATAAAAGCCCTTCCTTGTTGCATCAGCTACTATAAAA	Indel	3699	38.26%	
#85	TAAGCCTTCCTT------------------------------------GTTGCATCA	Indel	6560	71.27%	M
	TCGTCGTGGTGGTGAGATTAAGCCTTCCTTCCGCCTTATATAAACCCCCTCCAACCAGGTGCTAAGCACT	Intact	1410	15.32%	
#87	AGCCTTCCTTCCGCCTCACTAACATCAGCTACTATAAAAGCCCTTCCTTGTTGCATCAGCTACTATAAAA	Indel	4297	70.27%	M
	TCGTCGTGGTGGTGAGATTAAGCCTTCCTTCCGCCTTATATAAACCCCCTTC--CTTGTTGC-----ACT	Partial	1496	24.46%	
#91	AGCCTTCCTTCCGCCTCACTGGCAGATTCGTCGTGGTGGTGAGATTAAG--CCTTCC-------ACCATC	Partial	1597	10.63%	M
	AGCCTTCCTTCCGCCTCACTAACAACCAGGTGCTAAGCACTAACATCAGCTACTATAAAAGCCCTTCCTT	Partial	3320	22.10%	
	AGCCTTCC--ACCATCTCAAGGAGCT	Indel	10107	67.27%	
#94	TCGTCGTGGTGGTGAGATTAAGCCTTCCTTCCGCCTTATATAAACCCCCTCCAACCAGGTGCTAAGCACT	Intact	1247	15.73%	M
#99	TCGTCGTGGTGGTGAGATTAAGCCTTCCTTCCGCCTCACTAACACCCCCTCCAACCAGGTGCTAAGCACT	Partial	11084	52.91%	M
#101	TCGTCGTGGTGGTGAGATTAAGCCTTCCTTCCGCCTTATATAAACCCCCTCCAACCAGGTGCTAAGCACT	Intact	578	8.24%	M
	TCGTCGTGGTGGTGAGATTAAGCCTTCCTTCCGCCTCA----------CTAACATCAGGTGCTAAGCACT	Partial	4542	64.78%	
#102	TCGTCGTGGTGGTGAGATTAAGCCTTCCTTCCGCCTTATATAAACCCCCTCCAACCAGGTGCTAAGCACT	Intact	3898	48.88%	M
	AGCCTTCCTTCCGCCTCACTAACATCAGCTACTATAAAAGCCCTTCCTTGTTGCATCAGCTACTATAAAA	Indel	1090	13.67%	
#105	TCGTCGTGGTGGTGAGATTAAGCCTTCCTTCCGCCTTATATAAACCCCCTCCAACCAGGTGCTAAGCACT	Intact	2558	24.04%	M
#107	TCGTCGTGGTGGTGAGATTAAGCCTTCCTTCCGCCTTATATAAACCCCCTCCAACCAGGTGCTAAGCACT	Intact	14373	98.34%	B

Note: The line # number represents the unique hygromycin resistant transgenic event. Tissues from different tillers arisen from same T0 calli were pooled to collect DNA and subsequent genotyoing and deep-sequencing analysis. Some lines had mutliple editing events recovored from deep-sequence analysis as a result of pooling. Red colored bases indicate the EBE and green colored bases indicate

indels and/or substitutions and dashes represent deletions. The intact or partial EBE insertions with or withour indels are indicated along with number of reads and percentage of reads for the particular editing event. M indicates monoallelic and B indicates Biallelic as identified based on PCR-restriction digestion with *SexA*I or *Blp*I. * Percentage of WT reads not shown.

Supplementary Table 4: Whole genome Illumina re-seqeuencing sample information

Sample	Genotype	Total reads (million)	Read length	SRA accession
AG71-2	xa5	65.42	2 x 150 bp	SRR22904521
AG71-38	xa5	109.16	2 x 150 bp	SRR22904520
AG71-47	xa5	81.24	2 x 150 bp	SRR22904519
AG71-68	WT	76.96	2 x 150 bp	SRR22904518
AG62-47	$Xa23^{SW14}$	156.7	2 x 150 bp	SRR22904517
AG62-107-1	$Xa23^{SW14}$	110.19	2 x 150 bp	SRR22904516
AG62-107-2	$Xa23^{SW14}$	106.76	2 x 150 bp	SRR22904515
AG62-94	WT	72.14	2 x 150 bp	SRR22904514
Kitaake	WT	72.22	2 x 150 bp	SRR22904513

Supplementary Figures (1 – 5)

a

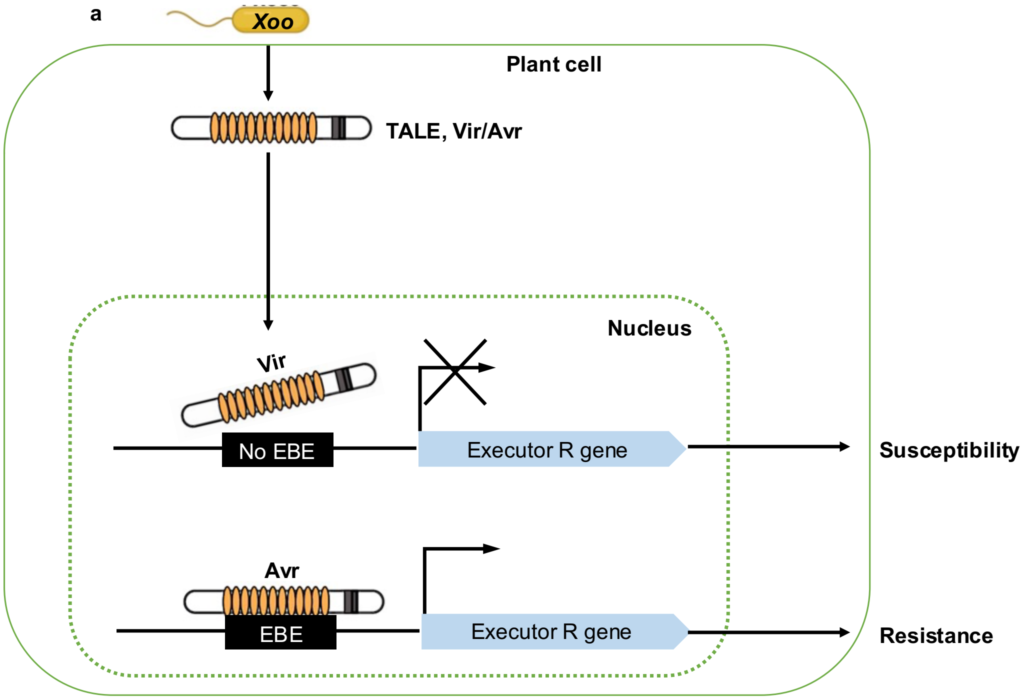

Executor R gene mediated dominant resistance mechanism

b

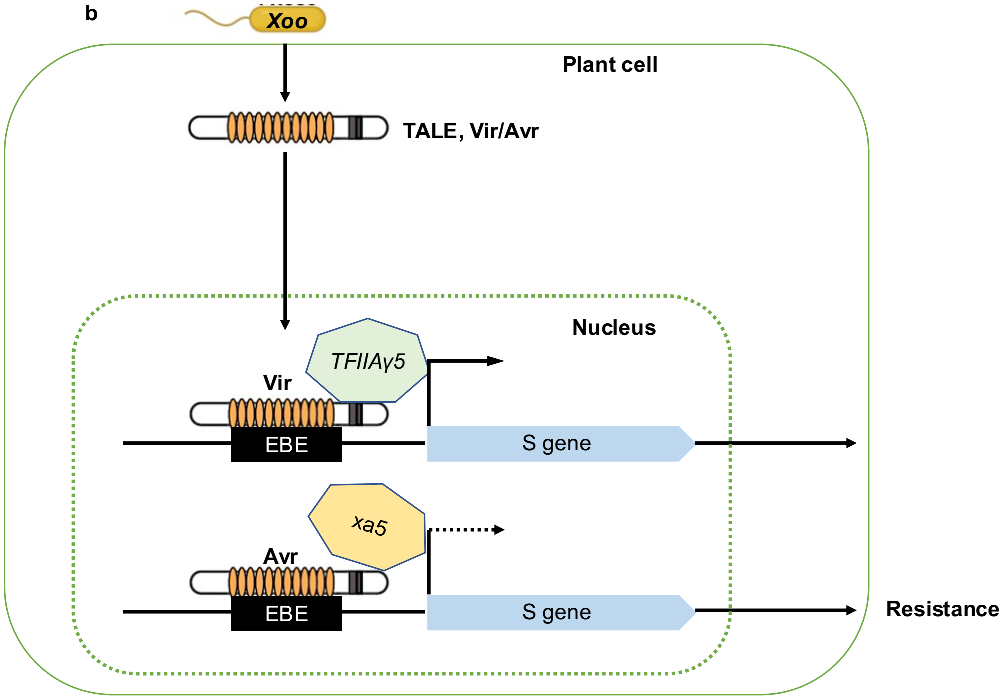

***xa5* mediated recessive resistance mechanism**

Suppl. Fig. 1. Schematic representation of *xa5* mediated recessive and executor *R* gene mediated dominant resistance mechanisms in rice. Upon infection, *Xoo* injects TALEs to plant cells via a Type Three Secretion System to colonize by transcriptionally activating the rice susceptibility genes. **a,** The dysfunctional executor *R* gene not carrying the EBE respective to the TALE does not get induced upon infection with *Xoo* and leads to susceptibility (crossed arrow). Functional executor R gene traps the TALEs into the EBE, gets induced and *R* proteins lead to hypersensitive response mediated resistance (solid arrow). **b,** The TALEs hijack rice's transcriptional machinery by recruiting the general transcription factor *TFIIAγ5* (green heptagon) to induce the susceptibility gene by binding at the EBE in promoter region (solid arrow). The V39E recessive resistant allele of *TFIIAγ5* termed as *xa5* (yellow heptagon) fails to interact with TALEs and induction of *S* genes could not reach a threshold of susceptibility, leading to resistance (dotted arrow). TALEs can act as a virulence (vir) or avirulence (avr) factor depending upon susceptible or resistant response in plants, respectively (**a**-**b**).

```
xa23 acatcagttacttccacc--acctcccatgtttcaaag----gt----aaattcgtcgtg  50
Xa23 ACATCAGCTACTATAAAAGTCCCTTCCGCGTCACTAACATCAGCTACTATAAAAGTCCC-  59
     ******* ****: .*..  .*** **. ** :*:**     *      *:*::.***

                         AvrXa7 EBE
                         PthXo3 EBE
               TATATAAACCCCCTCCAACCAGGTGCTAAG
xa23 gtggtgagattaagccttccttccgcctcactaacatcagctactataaaagcccttcct  110
Xa23 ----TTCCGAAACATCTTCCTCCCGCATCACTAACATCAGCTTCTATAAAAGCCCTTCCT  115
         * .  .::*.. ****** ****.***************:*****************

xa23 tgttgcatcatctcaaggagcttcaagcacttcctctctggcagcacttcctcatctcaa  170
Xa23 TGTTGCATCATCTCAAGGAGCTGCAAGCACTTCCTCTCTGGCAGCACTTCCTCATCTCAA  175
     ********************* **************************************

xa23 ggagttgcaaatg  183
Xa23 GGAGTTGCAAATG  188
     *************
```

Suppl. Fig 2. Promoter regions of *Xa23* (*xa23*) alleles for editing. Promoter sequences of two alleles (dominant *R* gene *Xa23* from CBB23 and recessive *xa23* from Kitaake) are used for alignment. Sequences with bars above are the overlapping EBEs for AvrXa7 and PthXo3 intended to knock in. Underlined sequence of *xa23* is the antisense sequence of protospacer for Cas9/gRNA with PAM in green and arrow showing nicking site. The shaded sequence of *Xa23* is the EBE of AvrXa23. Translation start triplets are in red.

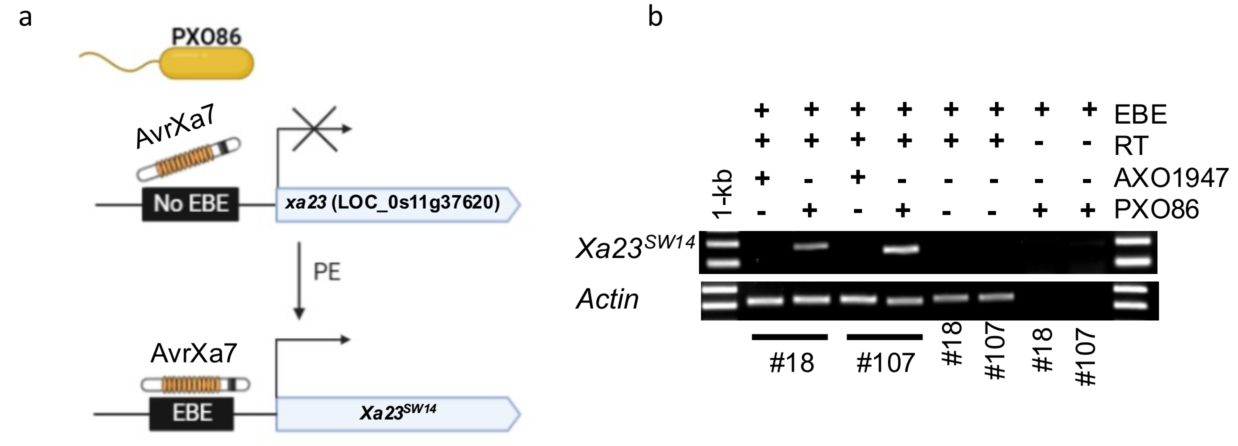

Suppl. Fig. 3. Induction of *Xa23^SW14^* by AvrXa7 carrying PXO86. **a**, Schematic model showing interaction of *xa23*/ *Xa23^SW14^* with *Xoo* TALE AvrXa7. *Xoo* strain PXO86 injects AvrXa7 inside plant nucleus. The dysfunctional *xa23* lacks the AvrXa7 EBE in the promoter region thus is not induced upon infection with PXO86 (crossed arrow). The prime edited functional allele *Xa23^SW14^* carries the AvrXa7 EBE and gets induced upon infection with PXO86 (arrow represents induction). **b,** Biallelic lines were tested for *Xa23^SW14^* induction using RT-PCR on RNA isolated from PXO86 and AXO1947 inoculated leaf tissues. AvrXa7 containing strain PXO86 was able to induce *Xa23^SW14^* expression while no induction was observed upon infection with AvrXa7 lacking strain AXO1947. Actin gene was used as an internal control and RNA without RT was used a DNA control. EBE, Effector binding element; RT, reverse transcriptase; 1-kb, DNA ladder.

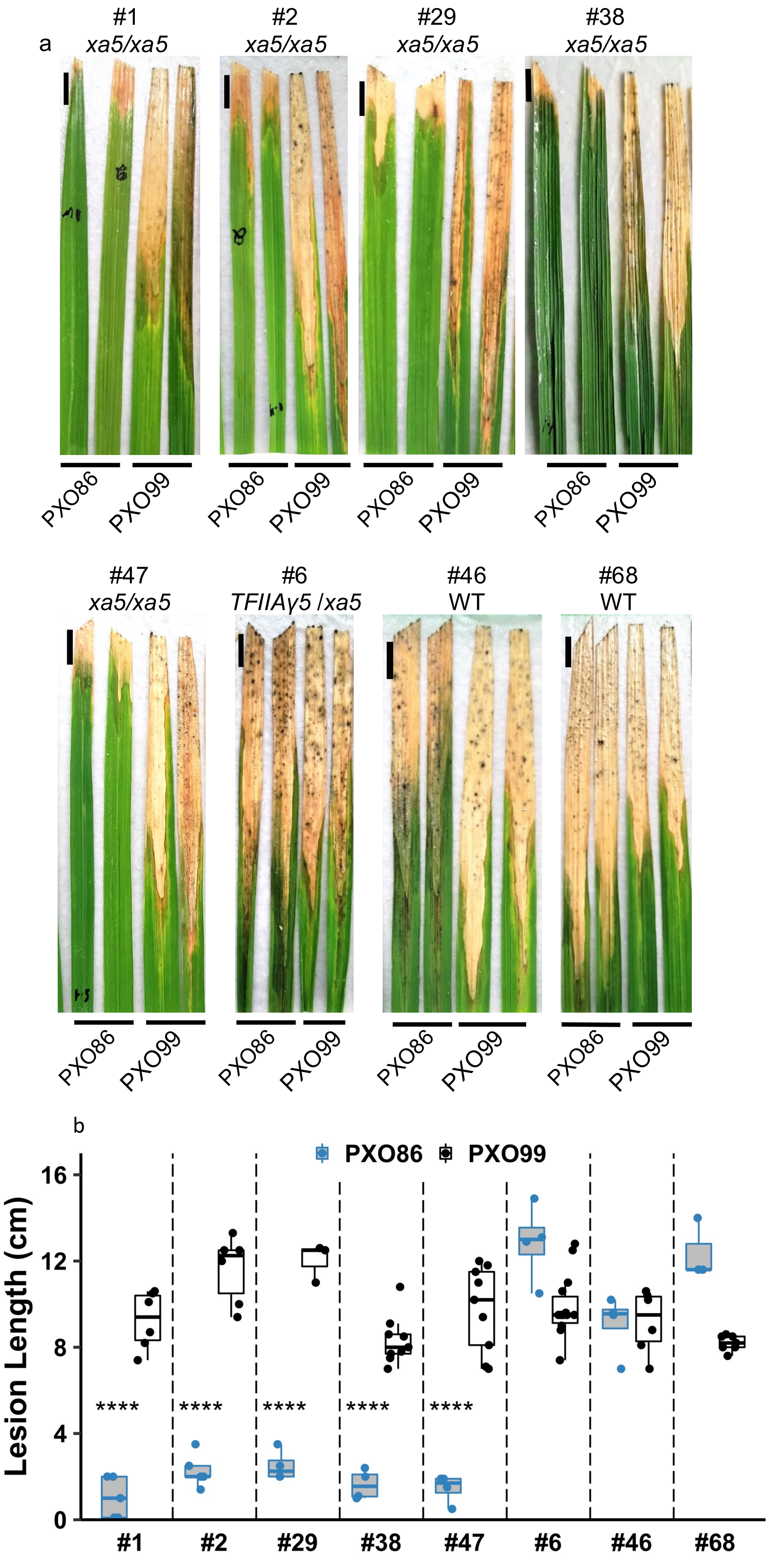
a
#1
xa5/xa5
#2
xa5/xa5
#29
xa5/xa5
#38
xa5/xa5
PXO86
PXO99
#47
xa5/xa5
#6
TFIIAγ5 /xa5
#46
WT
#68
WT
b
PXO86
PXO99
Lesion Length (cm)
16
12
8
4
0
****
#1
#2
#29
#38
#47
#6
#46
#68

Suppl. Fig. 4. Disease assay on *TFIIAγ5/xa5* edited biallelic, monoallelic, and WT lines. a, Pictures of leaves infected with PXO86 (carrying *avrXa7*) and PXO99 (harboring *pthXo1*) using leaf tip clipping method. Genotypes are labelled on top of each line. WT, Wildtype. **b**, Lesion lengths were measured 12 days post inoculation on three to five leaves of individual plants and plotted using R software. Scale bar; 1 cm. **** represents p-value < 0.0001 with t-test adjusted using Bonferroni correction for multiple comparisons.

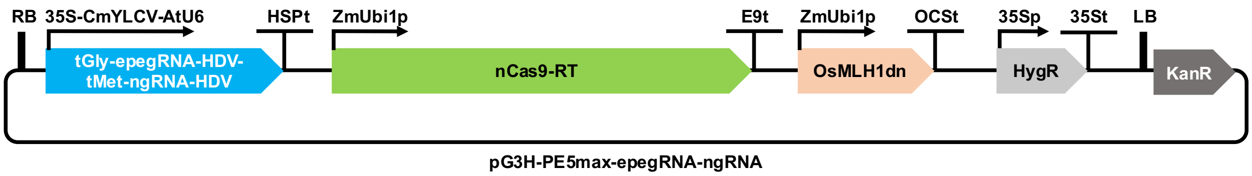

Suppl. Fig. 5. PE5max construct used for *Agrobacterium* mediated rice transformation. The engineered pegRNA and ngRNA system was driven by composite promoter system comprising 35S enhancer, Cestrum YLCV promoter, and Arabidopsis U6 promoter. nCas9-RT and OsMLH1dn were driven by maize Ubiquitin promoter. The pegRNA and ngRNA region was modified to target/edit either *TFIIAγ5/xa5* or *xa23/Xa23^{SW14}* resulting in pG3H-PE5max-TFIIAγ5/xa5 or pG3H-PE5max-xa23/Xa23^{SW14}, respectively. RB, right boarder; CmYLCV, Cestrum yellow leaf curling virus; AtU6, Arabidopsis U6; tGly, Glycine tRNA; epegRNA, engineered Prime Editing guide RNA; HDV, Hepatitis delta virus ribozyme; tMet, Methionine tRNA; HSPt, Heat shock protein terminator; ZmUbip; maize ubiquitin gene promoter; nCas9-RT, nickase Cas9 fused to reverse transcriptase. E9t, E9 terminator; OsMLH1dn, rice MLH1 domain; OCSt, OCS terminator; 35Sp, 35S promoter; HygR, hygromycing resistance gene; 35St, 35S terminator; LB, left boarder; KanR, kanamycin resistance gene.
